# Case of Dilatation of the Heart

**Published:** 1839-09-14

**Authors:** A. Stillé

**Affiliations:** Resident Physician to the Pennsylvania Hospital


// MEDICAL EXAMINER.

DEVOTED TO MEDICINE, SURGERY, AND THE COLLATERAL SCIENCES.

No. 37.] PHILADELPHIA, SATURDAY, SEPTEMBER 14, 1839. [Vol. II.

### CASE OF DILATATION OF THE HEART.

*Dyspnœa,—Præcordial Pain,—Irregular Pulse,—Œdema of Legs,—Death,—Effusion into Pericardium,—General Dilatation of Heart,—Lesions of Aortic and Mitral Valves.*

[Reported by A. Stillé, M. D., Resident Physician to the Pennsylvania Hospital.]

Daniel E——t, æt. 59, entered the Pennsylvania Hospital on the 23d of March, in a state of intoxication; unable to give distinct answers to questions; breathing with great apparent difficulty; an anxious expression of face, and coldness of the extremities. The following note of his examination was made:—In the præcordial region there appears to be dulness on percussion over a much larger space, and lower, than usual, viz., in a triangular area, the apex of which is on a level with the left nipple, and half an inch within it, and the base extending from the edge of the left costal cartilages, an inch below the sternum, to the articulation of the fifth rib with that bone. In this extent there is marked dulness; the impulse of the heart can scarcely be felt, except by a soft, undulatory tremor; its pulsations are, however, distinct in the epigastrium; the eye can detect no vibration of the parietes of the chest over the heart, unless at the moment of complete expiration, when a slight movement may be perceived at the sternal articulation of the fifth rib, and also an inch below the left nipple. There is no distinct prominence of the præcordial region; the respiration can be heard faintly all over it. The sounds of the heart are so varied in force, rhythm, and tone, that the ear cannot analyze them distinctly; a blowing and clapping sound may be distinguished, the one and then the other having by turns the greater intensity; the pulse is very irregular, and can with difficulty be counted, but about every tenth pulsation is forcible, the nine following gradually diminishing in strength till a perfect rest of about a second occurs; at the end of this, another series of pulsations, another interval, &c. There are about ninety pulsations in a minute. There is no pain near the heart, except in the epigastrium. The patient is not conscious of any palpitations, except after some violent exertion.

Over the whole chest, but notably behind, is heard a mucous rhonchus, fine in some points, and loose in others; anteriorly these sounds are mingled with sibilant and sonorous rhonchi. Percussion is very sonorous from the clavicles to the nipples on both sides, and posteriorly the sonorousness is equally good on both sides. No bronchophony nor blowing expiration in any point.

Blood was taken by cups from the præcordium, sinapisms applied to the extremities, and an ounce of castor oil, with fifty drops of laudanum administered.

*24th.* Patient enjoyed a much greater freedom in breathing after the application of the cups, and slept about half the night; bowels thrice opened without pain. The following note of his condition was taken on the morning of the 24th. Decubitus dorsal; skin dark brown; muscles developed and firm; hair scanty, brown, grizzled; eyes blue; expression calm, rather dull; right pupil more contracted than left, and more sensible to the impression of the light; no cephalalgia; sight and other senses unimpaired; intelligence inferior, but apparently natural; right corner of mouth drawn slightly outward; tongue straight, steady, moist, a little furred; deglutition easy; no appetite; no colics; abdomen supple, without any tumour; no pain but at epigastrium, where it is less than yesterday; urine free, of dark yellow colour, but lighter than it was; skin moist, of moderate warmth; pulse 90; slight œdema of feet and legs. Respiration 20 to 25, not deep; the chest dilates suddenly, and as if of one piece; it contracts rapidly; the intercostal spaces evidently depressed, during inspiration, between second and third ribs; the muscles of the neck take an active part in the mechanism of inspiration. Paroxysms of coughing several times in the day, with expectoration of firm albuminous sputa in a thin, clear liquid; auscultation and percussion in all points as yesterday. Patient can give no distinct account of his history, but says he has had shortness of breath for about three years.

Blister applied to præcordium; low diet; barley water; absolute rest.

*26th.* Free discharge from blistered surface; patient declares himself greatly relieved; he breathes more easily; expression of distress diminished; pulse as yesterday; no auscultation of heart on account of blister. The visiting physician, Dr. Wood, prescribes,

℞. Calomel. gr. i.

P. Opii, gr. ¼.

P. Ipecac. gr. ii. Sumend. ter in die.

*27th—30th.* No remarkable change in the respiration, nor in the appreciable state of the heart: expectoration of thin, frothy liquid, containing some viscid, rusty sputa. Prostration marked, particularly after talking; decubitus dorsal; skin has a yellowish tinge, as well as the sclerotica; copious sweats; occasional vomiting; three or four stools a-day, of moderate consistence; appetite very slight. Calomel and opium continued; ipecacuanha suspended.

*May 1st.* Prostration more considerable; expression less lively; cannot speak without getting out of breath; intelligence and senses perfect; no vomiting; stools doubtful; tinge of sclerotica deeper; pain to right of spine, an inch below inferior angle of scapula; *percussion rather less resonant on right than on left side, below the spine of scapula; over same space a mucous rhonchus, fine at the root, and looser near the base of the lung;* on left side a mucous rhonchus, looser than on opposite side. Anteriorly, sonorousness on percussion great down to nipples on both sides; respiration vesicular. The sounds of the heart are now more easily analyzed; *the first is rude, but without distinct blowing; the second is sharp, shrill, and clapping.* The rhythm of the heart's motions is not natural, and corresponds to that of the pulse noted April 23d.

Moderate œdema of legs as high as knees; no evident tumour of abdomen, or distinct signs of effusion; urine high coloured; extremities cool; skin of trunk warm and clammy; pulse feeble, and so irregular as not to be accurately counted.

6½, *P. M.* Patient refuses to take brandy punch offered him; says that he loathes it, and that his time is come; prostration extreme; moaning; complains of pain in back and right side; pulse very feeble; sinapism to right side.

*2d.* E. continued to make much noise by his lamentations all night; this morning he sat up while his bed was making; asked for water, which the nurse went to procure him; when the nurse returned, he was dead.

*Autopsy of E——t,* twenty-four hours after death. Weather cool and clear; general colour of body yellowish; marbled; veins of surface distinct; rigidity marked; emaciation slight; some œdema of both legs and feet; percussion of præcordium gives same results as during life; subcutaneous fat considerable; muscles of dull red, well developed. *Abdomen* contained about one and a half pints of dark coloured fluid; no adhesions of peritoneum, no false membrane; right kidney about four inches, left five inches long; cortical substance very much developed; distinct from tubular, and filled with small yellow and red points; bladder contracted and empty; stomach about ten inches long, partially filled with whitish, sour smelling fluid; no injection of its mucous membrane, which is pale, and of good consistence; same contents and appearance of small intestine; cœcum contains a little mucus, membrane pale and sound. Liver very firm, even hard, presenting two substances with evident difference; one of a dark reddish-brown colour, and the other of a clear yellow in points; the gall bladder distended with dark, but thin bile. *Chest.* *Left lung* adherent in patches posteriorly, and to diaphragm; no effusion into its pleural cavity; anterior portion remarkably light and crepitant, without notable development of cells; tissue there exceedingly tough; both lobes permeable to air, but rather less so near spine and posterior part of base; frothy mucus exudes on pressure; in the points just mentioned, the lung is redder and heavier, and less firm, but still resistant; no pus, no tubercles; bronchiæ contain small quantity of whitish mucus, their lining membrane thick, pale, puffed, and corrugated longitudinally. *Right* pleural cavity contains about half a pint of yellowish serum; lung adheres firmly behind in middle third; on removing it there is found between the pulmonary and costal false membranes, a jelly-like substance, (which I take to be serum in the inter-pleural cellular tissue,) about three lines thick; the same appearance, though somewhat less, on left side; anterior portion of lung as on left side; the posterior, near the spine and at base, is less permeable to air than on left side; its colour is deeper, the tissue is more friable, and its section clearly granular; bronchiæ precisely as on left side.

*Brain,* offers no appreciable lesion of any kind whatever.

*Spleen,* small, shrivelled and firm.

*Heart.* Pericardium contains about six ounces of turbid fluid, of a reddish-yellow; the pericardium is of a dull red colour, without distinct capillary injection; its surfaces smooth, free, and without deposit. The heart weighs 3 lb. 11 oz. apothecaries' weight. Colour of heart externally, pale red; the course of its superficial veins much loaded with fat; its tissue is flabby, and, when laid upon the table, flattens itself remarkably; in this position, its greatest breadth measures four and a half inches, (Fr.;) its length, from apex to nearest point of pulmonary artery, four and a half inches, (Fr.;) the cavities all contain black clots. *The right ventricle* is of a brownish-red colour, its surface smooth and shining, and the maximum thickness of its walls two lines, exclusive of the *columnæ carneæ,* which are but little developed, and very loose in texture. The circumference of this cavity measures five inches; its length four and a half; circumference of pulmonary artery at free edge of valves, two and three quarters inches; these latter are free, smooth, and transparent; the lining membrane of pulmonary artery is of a light red colour; the tricuspid valves are of the same hue, and contain a few points of cartilaginous hardening.—*The right auricle:* parietes very thin, capable of easily containing a large hen's-egg; lining membrane as in ventriculo-muscular columns, developed and in general parallel to one another.—*Left ventricle:* five inches in circumference; length three and three quarters; greatest thickness of parietes, eight lines; at apex, (least,) two lines; columnæ carneæ, few and small; colour of interior, reddish-brown; lining membrane smooth and transparent. Mitral valve very small, incomplete, particularly in its usual attachment to the edge of the *osteum venosum,* and slightly rigid. Aortic valves opaque, of bright red colour, thickened and cartilaginous about their attached edges, with numerous points of cartilage disseminated over their whole surface, partially but not completely stiffening them. The aorta is studded for several inches with cartilaginous and ossific deposites, chiefly near the valves, and in masses of irregular form, generally about the size of a millet seed, and never above the general surface of the artery. At the free edges of the valves the circumference is two and a half inches. The interior of the left ventricle is perfectly smooth; the auriculo-ventricular orifice is completely surrounded and contracted by a pad or thickening of cartilaginous matter, about two lines in thickness, and very hard towards the anterior surface of the heart; it is one and a half lines thick, and softer posteriorly; on the ventricular side of this formation, the cordæ pectineæ are attached, and they are themselves stiff and semi-cartilaginous. On the auricular side of the ridge just described, and towards the *foramen ovale*, there is a deposite of gritty, calcareous matter, seven lines long by four broad; one-half of this space is still almost covered by the endocardium; the other is open, excavated to the depth of one line, and has projecting from it, so as to overhang the auriculo-ventricular orifice, a rough granular mass of stony matter, irregular in shape, and brittle, measuring three lines long, by one and a half thick.

This case has been reported in considerable detail, not so much from the general interest belonging to it, as because it is the only one, within the reporter's knowledge, of a calcareous excrescence on the auricular side of the *osteum venosum*. Diligent search has been made amongst the standard works on diseases of the heart, as well as in the periodical publications of several years past, without the discovery being made of any history of a similar lesion; indeed, most of the authors, after describing the various alterations of the mitral valve, remark, that while these are frequent, the lesions of the left auricle near that valve, and the orifice it closes, are exceedingly rare.

